# The three-dimensional easy morphological (3-DEMO) classification of scoliosis, part II: repeatability

**DOI:** 10.1186/1748-7161-1-23

**Published:** 2006-12-21

**Authors:** Alberto Negrini, Stefano Negrini

**Affiliations:** 1 ISICO (Italian Scientific Spine Institute), Milan and Fondazione Don Carlo Gnocchi IRCCS-ONLUS, Milan, Italy

## Abstract

**Background:**

In the first part of this study we proposed a new classification approach for spinal deformities (3-DEMO). To be valid, a classification needs to overcome the repeatability issue which is inherent both in the used classificatory system and in the measured object.

**Aim:**

The aim of this study is to present procedures and results obtained within the repeatability of 3-DEMO classification for scoliosis analysis.

**Method:**

We acquired the data of 100 pathological and 20 normal spines with an optoelectronic system (AUSCAN) and of two dummies with simulated spine deformity. On the obtained 3D reconstruction of the spine, we considered the coronal view with a spinal reference system (Top View) and its three related parameters, defined in part I, constituting the 3-DEMO classification. We calculated the repeatability coefficient for the subjects (two acquisitions for each subject with a time interval of 26 ± 12 sec), whereas we evaluated the system measurement error calculating the standard deviation of 50 consecutive acquisitions for each dummy.

**Results:**

Comparing the results of the two types of acquisition, it emerged that the main part of parameters variability was due to postural adjustments The proportion of agreement for the 3-DEMO parameters gives a k value above 0.8; almost 10% of patients changed classification because of postural adjustments, but none had a "mirror-like" variation nor a change in more of one parameter at a time Repeatability coefficient is lower than the previously calculated normative limits.

**Discussion:**

The 3-DEMO classification has a high repeatability when evaluated with an optoelectronic system such as the AUSCAN System, whose systematic error is very low. This means that the implied physiological phenomenon is consistent and overcomes the postural variability inherent in the measured object (normal or pathological subject).

## Background

The third dimension today is a clinical problem to be solved every time surgery [1-3], bracing [4,5], or exercises are proposed [6,7], but today clinicians lack tools to three-dimensionally understand the scoliotic spine, partly because of complexity, costs and reduced diffusion of involved instruments, but also because the existing proposed classifications [8,9] are very complex and born mainly outside the clinical field. Efforts to clinically face the third dimension, mainly for surgical purposes, have been done with new classifications [10] that anyway are mainly bidimensional. In the first part of this study [11], we proposed a 3D clinical classification of spine morphology projected on to the horizontal plane (3-DEMO), the "Top View": this constitutes a projection on to an auxiliary plane that seems optimal for the comprehension of scoliotic spine third dimension. In this plane, the trend of the curves in antero-posterior and latero-lateral projections can be simultaneously viewed and only the information relating to the vertical axis is lost. We used an optoelectronic system (AUSCAN) to obtain a 3D spine reconstruction, whose repeatability had been evaluated in the past [12]. An expert clinician evaluated the morphological reconstruction of 149 pathological spines to find parameters that could be used for classificatory ends: Direction, Shift and Phase were defined and were verified both in a mathematical way and through computer simulations.

For a classification to be valid, it is necessary to evaluate its stability by examining the parameters variation on which this classification is based. The adoption of an optoelectronic device like the AUSCAN system guarantees a very high precision [12]: system error is less than 1 mm; unlike typical devices used for the evaluation of a patient with spinal deformities, this non-ionizing system permits to repeat the acquisitions without risks for the subjects; it returns three-dimensional data about the spine; it allows to evaluate the dynamic aspect of the posture [13]. This last point is particularly important, because the use of a ionizing instrumentation does not permit to evaluate the incidence of postural variability on the parameters used for Ponseti classification and for Cobb angles calculation [14]. According to the previously proposed classification for error sources of the AUSCAN System Analysis [12], we focused on System error and on in vivo repeatability of the phenomenon, knowing that the latter includes the former [12]. We designed a protocol in order to define the quantitative criteria used for the 3-DEMO classification [11]. We were interested in evaluating the repeatability of the 3-DEMO classification, i.e. the classification in the single subject, not the repeatability of the method used to obtain it, because this classification can be obtained with many other methods, both ionizing and not. According to the System adopted to pursue the 3-DEMO classification, in the future it will be necessary to verify the repeatability of each measuring device. The aim of this study is to present procedures and numerical results regarding the repeatability of 3-DEMO classificatory parameters.

## Materials and methods

### Population

We included in our study 20 normal subjects [11]: 16 females and 4 males, with a mean age of 14.6 ± 2.0, weight and height of 49.9 ± 10.0 and 160.7 ± 13.1 respectively. Moreover we considered 100 subjects (75 females) affected by scoliosis and/or hyperkyphosis, who entered one of our institutes (FDCG) between January 1990 and January 1996 for treatment. Mean age was 16.2 ± 2.8, while weight and height were 53.6 ± 13.8 and 163.1 ± 9.8 respectively. Table 1 gives the patients' radiographic characteristics.

**Table 1:** Radiographic data of studied population.

	**Sample**	**Cobb Degrees (mean ± S.D.)**
**Single scoliosis**		
Right thoracic	11	45 ± 10
Left thoracic	2	45 ± 4
Right thoraco-lumbar	4	47 ± 8
Left thoraco-lumbar	2	21 ± 12
**Double scoliosis**		
Right thoracic	30	40 ± 11
Left lumbar		37 ± 11
Left thoracic	5	29 ± 13
Right lumbar		38 ± 13
Right thoracic	23	32 ± 11
Left thoraco-lumbar		38 ± 15
Left thoracic	6	31 ± 11
Right thoraco-lumbar		39 ± 15
**Triple scoliosis**		
Right thoracic	1	13
Left thoracic		19
Right lumbar		27
**Hyperkyphosis**	16	Kyphosis: 67 ± 9
		Lordosis: 58 ± 13

### Data acquisition

Data have been acquired with AUSCAN system, while Top View and related parameters have been calculated as described in the first part [11]. In order to quantify the error due to the AUSCAN system, we made 50 acquisitions on 2 dummies with simulated spinal deformities of different types. The characteristics of the two dummies are reported in Table 2. For each acquisition, the same parameters used for the subjects have been calculated. To evaluate the repeatability due to subjects postural adjustments, we acquired the data twice in scoliosis subjects and in normals with time intervals between the acquisitions of 26 ± 12 sec and 22 ± 6 sec respectively. We were interested in classification repeatability in the subject, not in the method used to obtain it. Thus, in order to avoid the variability due to markers repositioning on the subject and of subject repositioning in front of the cameras, they were asked to remain in the same standing position during data acquisition. This implies that variability sources are only the AUSCAN System measurement error and the postural adjustments of the subjects [12].

**Table 2:** Spinal morphologies of the two dummies according to 3-DEMO: mean (mm) and standard deviations (mm) of the 50 acquisitions are listed. The first dummy was classified as a right curve, backward/left shifted, whereas the second one as a left curve, anisophasic, forward shifted.

**Dummy**	**Direction (°)**	**Lateral shift (mm)**	**Sagittal shift (mm)**	**Phase**
1	16.6 ± 0.11	-3.9 ± 0.05	-14.7 ± 0.10	1.7 ± 0.06
2	-18.8 ± 0.51	0.3 ± 0.03	6.9 ± 0.17	13.6 ± 0.09

### Statistical analysis

The measurement system variability has been evaluated by means of the standard deviation of the parameters, calculated basing on the 50 acquisitions on dummies. Subjects variability has been evaluated through the repeatability coefficient [15], that is today considered as the gold standard for this evaluation. The repeatability coefficient is calculated as twice the standard deviation of the values differences between the first and the second acquisition, and represents the 95% Confidence Interval of the distribution of those differences. Scoliosis group classification agreement between the two acquisitions has been evaluated with the k coefficient, the gold standard for this evaluation; this has not been done in the normal group due to the small sample considered.

## Results

The incidence of measurement error due to the AUSCAN System on the 3-DEMO parameters evaluated on dummies (Table 2) is very low: the standard deviation is always below 0.51 mm, and most of the time it remains below 0.1 mm. In vivo, the repeatability coefficient is almost 10 times the AUSCAN System error (Table 3), and this value is doubled in the normal sample for both Shift parameters, while it remains the same for Direction and Phase. The proportion of agreement for the 3-DEMO parameters (Table 4) gives a high k value, above 0.8; almost 10% of patients (and less in the normal group) changed classification because of postural adjustments, but none had a "mirror-like" variation (i.e. from left to right curve, or from forward to backward translation), nor a change in more of one parameter at a time.

**Table 3:** Repeatability analysis of the parameters in patients' acquisitions: mean difference and repeatability coefficients.

	**Direction (°)**	**Frontal shift (mm)**	**Sagittal shift (mm)**	**Phase**
**Scoliosis group**				
Mean difference	0.02	0.01	0.46	0.02
Repeatability Coefficients	7.8	2.4	5.0	2.8
**Normal group**				
Mean difference	1.2	0.9	6.7	0.1
Repeatability Coefficients	9.0	5.2	19.6	2.1

**Table 4:** Rates of 3-DEMO classification changes for the various considered parameters.

	**Direction (°)**	**Frontal Shift (mm)**	**Sagittal Shift(mm)**	**Phase**
**Scoliosis group**				
Same classification	90%	90%	91%	90%
Changed classification	10%	10%	9%	10%
k	0.85	0.85	0.87	0.81
**Normal group**				
Same classification	100%	100%	90%	95%
Changed classification	0%	0%	10%	5%

## Discussion

When considering a new classification of pathological processes, it is determinant to be sure that it registers pathological parameters that are stable at a short-term. Another future problem will be to monitor changes due to disease treatment and/or progression. Conceptually, there is a distinction between the method used to obtain the data, and the data themselves. The "rumour" of the measuring system must be lower than the one of the pathological process itself. In scoliosis field, where radiographic measurements are considered the gold standard, the measuring system error is classically evaluated through intra- and inter-observer variations in Cobb measurements [16,17]. Even if usually ignored and scarcely considered, in vivo we also find postural and repositioning errors [14,13] as well as circadian variations [18]. In this paper we evaluated both the stability of 3-DEMO as an evaluation tool for spinal deformities and the measurement system errors, to be sure that the latter does not overcome the former.

The variations due to the measurement device are much lower than those due to the subjects: this implies that the error for the subjects is mainly due to postural adjustments. Posture is a phenomenon that should always be carefully considered when looking at patients with scoliosis: it has been studied in the past as the variation between standing and supine radiographs [19,20], but we must carefully consider that posture is not static. It can dynamically and continuously cause variations in standing position, that have consequences in all evaluations performed on scoliosis subjects [13,14]. Postural variations do not significantly change 3-DEMO parameters.

The repeatability coefficient should be used as a limit for the parameters of the 3-DEMO, supposing that they overcome normative parameters [11], but we verified that this is not true (Table 5). So, their main significance is in evaluating single patients, whose classificatory values are closer to normative limits than to the repeatability coefficient value: in these cases the 3-DEMO parameter could change when repeating the evaluation and should be carefully considered.

**Table 5:** Repeatability coefficients of 3-DEMO are lower than normative data.

**Parameter**	**Repeatability Coefficient**	**Normative limits**	
		**Lower limit**	**Higher limit**

Direction	7.8	-9.2	13.1
Frontal Shift	2.4	-8.0	4.1
Sagittal Shift	5.0	-26.4	4.6
Phase	2.8	8.1	

## Conclusion

The new 3-DEMO morphological classification has a high repeatability when evaluated with an optoelectronic system such as the AUSCAN System, whose systematic error is very low. This means that the implied physiological phenomenon is consistent and overcomes the postural variability inherent in the measured object (normal or pathological subject). If in the future alternative methods will be developed to be applied in everyday clinical usage (studies with this aim are already under way), the repeatability of each single method needs to be assessed.
